# Disruption of *Drosophila melanogaster* Lipid Metabolism Genes Causes Tissue Overgrowth Associated with Altered Developmental Signaling

**DOI:** 10.1371/journal.pgen.1003917

**Published:** 2013-11-07

**Authors:** Takeshi Sasamura, Kenji Matsuno, Mark E. Fortini

**Affiliations:** 1 Department of Biochemistry and Molecular Biology, Thomas Jefferson University, Philadelphia, Pennsylvania, United States of America; 2 Department of Biological Science, Osaka University, Machikaneyama, Toyonaka, Osaka, Japan; University of Cambridge, United Kingdom

## Abstract

Developmental patterning requires the precise interplay of numerous intercellular signaling pathways to ensure that cells are properly specified during tissue formation and organogenesis. The spatiotemporal function of many developmental pathways is strongly influenced by the biosynthesis and intracellular trafficking of signaling components. Receptors and ligands must be trafficked to the cell surface where they interact, and their subsequent endocytic internalization and endosomal trafficking is critical for both signal propagation and its down-modulation. In a forward genetic screen for mutations that alter intracellular Notch receptor trafficking in *Drosophila melanogaster*, we recovered mutants that disrupt genes encoding serine palmitoyltransferase and acetyl-CoA carboxylase. Both mutants cause Notch, Wingless, the Epidermal Growth Factor Receptor (EFGR), and Patched to accumulate abnormally in endosomal compartments. In mosaic animals, mutant tissues exhibit an unusual non-cell-autonomous effect whereby mutant cells are functionally rescued by secreted activities emanating from adjacent wildtype tissue. Strikingly, both mutants display prominent tissue overgrowth phenotypes that are partially attributable to altered Notch and Wnt signaling. Our analysis of the mutants demonstrates genetic links between abnormal lipid metabolism, perturbations in developmental signaling, and aberrant cell proliferation.

## Author Summary

The development of complex, multicellular animal tissues requires the coordinated function of many different cell-cell communication pathways, in which secreted or cell-surface-anchored ligands from one cell typically activate a receptor on the surface of other cells, which in turn regulates downstream gene transcription and other cellular processes. We used a genetic approach in the fruit fly *Drosophila melanogaster* to search directly for mutations that perturb intracellular trafficking of a major signaling receptor, namely the Notch receptor, which controls cell differentiation in various tissue contexts. The Notch signaling pathway, like other key developmental signaling pathways, is evolutionarily conserved and functions in a similar manner in *D. melanogaster* and mammals, including humans. We recovered and characterized mutations in two genes that encode different enzymes involved in cellular lipid metabolism. Both mutants alter not only Notch signaling but also downstream activity of another highly conserved signaling pathway mediated by the Wingless protein, illustrating that alterations in cellular enzymes of lipid metabolism can exert complex effects on multiple critical signaling pathways. We also found that the new mutants exhibit dramatic cell overproliferation effects, reinforcing findings from mammalian studies suggesting that lipid metabolism might play an important role in oncogenesis and tumor progression.

## Introduction

Developmental patterning in metazoans requires the coordinated activity of several intercellular signaling pathways. In *D. melanogaster*, Notch and Wnt signaling are critical for the formation of diverse organs and tissues, and both pathways also regulate cell proliferation and apoptosis during development [1]–[4]. Notch signaling is activated by binding of DSL ligands to the Notch receptor, which induces proteolytic cleavage of Notch by ADAM/TACE metalloproteases and subsequent cleavage by gamma-secretase to generate the Notch intracellular signaling fragment NICD [1], [2]. NICD translocates to the nucleus where it regulates target gene expression by displacing co-repressors from transcriptional complexes and converting them into active complexes [5], [6].

Notch signaling is strongly modulated by additional posttranslational mechanisms, including glycosylation, ubiquitylation, and endosomal trafficking [2], [7]. Endocytosis of Notch and its ligands in both signal-receiving and signal-sending cells is required for productive signaling [8], generating tensile forces needed to expose the ADAM/TACE cleavage site in Notch and facilitate receptor proteolysis [9]. Several mutants with impaired trafficking of ligand-activated Notch are associated with reduced signaling, while others that perturb trafficking of non-activated Notch exhibit Notch hyperactivation and tissue overgrowth [10]–[14]. Intracellular Notch trafficking thus regulates both signal activation and the degradation of inactive Notch receptors that might otherwise contribute to inappropriate signaling. Moreover, this endocytic regulation of Notch signaling is strongly influenced by its membrane lipid microenvironment; mutations in *D. melanogaster phosphocholine cytidylyltransferase* alter Notch endosomal routing and activation [15] and mutations in *D. melanogaster alpha-1,4-N-acetylgalactosaminyltransferase-1* affect endocytosis and potency of the Notch ligands Delta and Serrate [16].

Canonical Wnt/Wingless signaling is similarly needed for a diverse array of tissue patterning processes and is also influenced by membrane trafficking [3], [4]. Wnt, a secreted ligand, binds to receptors of the Frizzled and LRP/Arrow families, leading to recruitment of Disheveled and stabilization of ß-catenin/Armadillo. Accumulation of ß-catenin/Armadillo in turn triggers downstream gene activation through TCF transcription factors [17], [18]. Wnt proteins are modified by lipid attachment [19], [20], and endocytosis limits secreted Wnt diffusion and modulates intracellular signaling [4], [21].

To identify new genes required for trafficking of developmental signaling molecules, we performed a forward genetic screen for mutations that alter the intracellular accumulation of Notch in developing *D. melanogaster* wing tissues. Among ∼40 new mutants recovered, two mutants displayed a strikingly similar phenotype of abnormal, non-cell-autonomous accumulation of Notch, Wingless, and other membrane proteins in endosomes and lysosomes. These two mutants disrupt essential enzymes of lipid metabolism, serine palmitoyltransferase (SPT) and acetyl-CoA carboxylase (ACC), which are encoded by the *lace* and *ACC* genes, respectively. SPT catalyzes the covalent attachment of serine to long chain fatty acyl-CoA to produce 3-ketodihydrosphingosine during sphingolipid biogenesis [22], [23]. Sphingolipids are major constituents of lipid rafts, a specialized membrane microdomain involved in endocytosis and signaling [24], [25]. In addition, bioactive sphingolipids participate in various signaling events, regulating cell proliferation, differentiation, apoptosis, and other cellular functions [26]. ACC catalyzes the carboxylation of acetyl-CoA to produce malonyl-CoA in the *de novo* synthesis of fatty acids [27], [28], which are needed for various cellular functions including energy storage, membrane biogenesis, and serving as precursors for phospholipid biosynthesis.

Notably, the *D. melanogaster* SPT and ACC mutants also exhibit tissue overgrowth phenotypes indicative of effects on cell proliferation. Analysis of downstream targets of Notch and Wingless reveals that this overgrowth is likely to involve modulatory effects on these pathways, reflecting both Notch hyperactivation and impaired Wingless signaling. Epistasis studies demonstrate that the overgrowth partially depends upon the Notch effector Su(H) as well as gamma-secretase function, and can also be partially suppressed by activated Armadillo, confirming that both Notch and Wingless dysregulation contributes to the *lace* and *ACC* mutant overgrowth phenotypes. Our findings emphasize the importance of lipid metabolism for establishing and maintaining the membrane compartments in which key developmental signaling pathways operate, and illustrate how general metabolic processes can exert complex, pleiotropic effects on multiple pathways needed for tissue growth and patterning.

## Results

### Abnormal Notch trafficking in *D. melanogaster lace* and *ACC* mutants

To identify new genes required for proper Notch trafficking, we designed a forward genetic screen in which homozygous mutant tissue is directly examined for aberrant Notch accumulation using antibody immunoanalysis. Because important trafficking genes would likely encode products essential for organismal viability, we created clones of homozygous mutant tissue in developing imaginal wing discs of otherwise heterozygous *D. melanogaster* using the FLP-FRT mosaic method [29] (see Materials and Methods). This approach also allows mutant tissues to be compared directly to adjacent heterozygous tissue in each sample, eliminating variability in fixation time, antibody penetration, and other parameters. Following screening of 3335 mutagenized second chromosome arms, we identified over 40 genes which, when mutated, alter the pattern of Notch trafficking as visualized using an antibody directed against the Notch intracellular domain.

**Figure 1:**
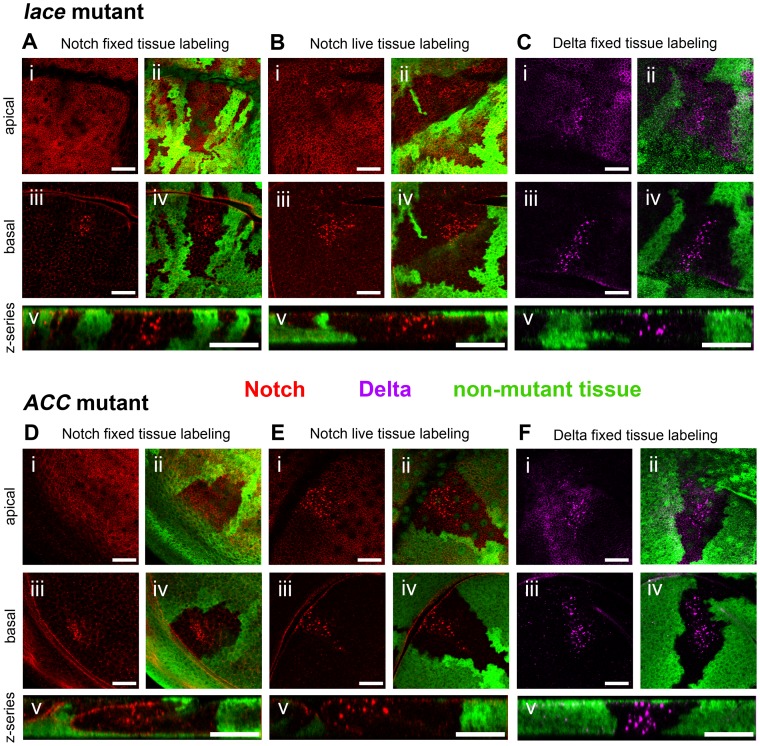
Notch and Delta accumulate abnormally in *lace* and *ACC* mutant tissues. Confocal optical sections through *D. melanogaster* wing imaginal discs bearing homozygous mutant clones of *lace^18^* (A–C) or *ACC^1^* (D–F) showing accumulation of Notch (A, D) and Delta (C, F) in fixed tissue samples, and endocytic internalization of Notch in live tissue samples (B, E). Areas devoid of GFP marker gene expression (green) correspond to mutant cell regions. Each set of five images (i–v) depict an apical (i, ii) and basal (iii–iv) horizontal section showing Notch (red in A, B, D and E) or Delta (magenta in C and F) accumulation, the same images overlaid with corresponding GFP expression to indicate clone locations (ii and iv), and a representative z-series showing the distribution of Notch or Delta along the apicobasal axis of the disc tissue (v). Scale bars, 20 µm.

Two lethal mutants, subsequently identified as *lace* and *acetyl-CoA carboxylase* (*ACC*) mutants, exhibited similar effects on Notch trafficking in homozygous mutant clones. In both cases, mutant cells display large, abnormal intracellular Notch-positive vesicles confined to internal clone regions approximately 3–4 cell diameters away from clone boundaries (Figures 1A and 1D). In homozygous mutant cells near clone boundaries, mutant effects on Notch trafficking are evidently rescued by non-cell-autonomous activity provided by nearby wildtype cells. The *D. melanogaster* wing disc is an oriented columnar epithelial monolayer, and the aberrant Notch-containing vesicles are observed throughout mutant cells from basement membrane to apical surface, in contrast to the predominantly apical accumulation of Notch in wildtype cells (Figures 1A and 1D). This non-autonomous Notch trafficking defect is consistently observed in both newly isolated alleles of *ACC* (*ACC^1^* and *ACC^2^*), both newly isolated alleles of *lace* (*lace^18^* and *lace^19^*), and a previously isolated amorphic allele of *lace* (*lace^2^* [30]) (Figures 1A and 1D; Supplemental Figure S1A–E). In the *lace* and *ACC* mutants, the Notch ligand Delta is similarly mislocalized in a non-cell-autonomous manner (Figures 1C and 1F).

**Figure 2:**
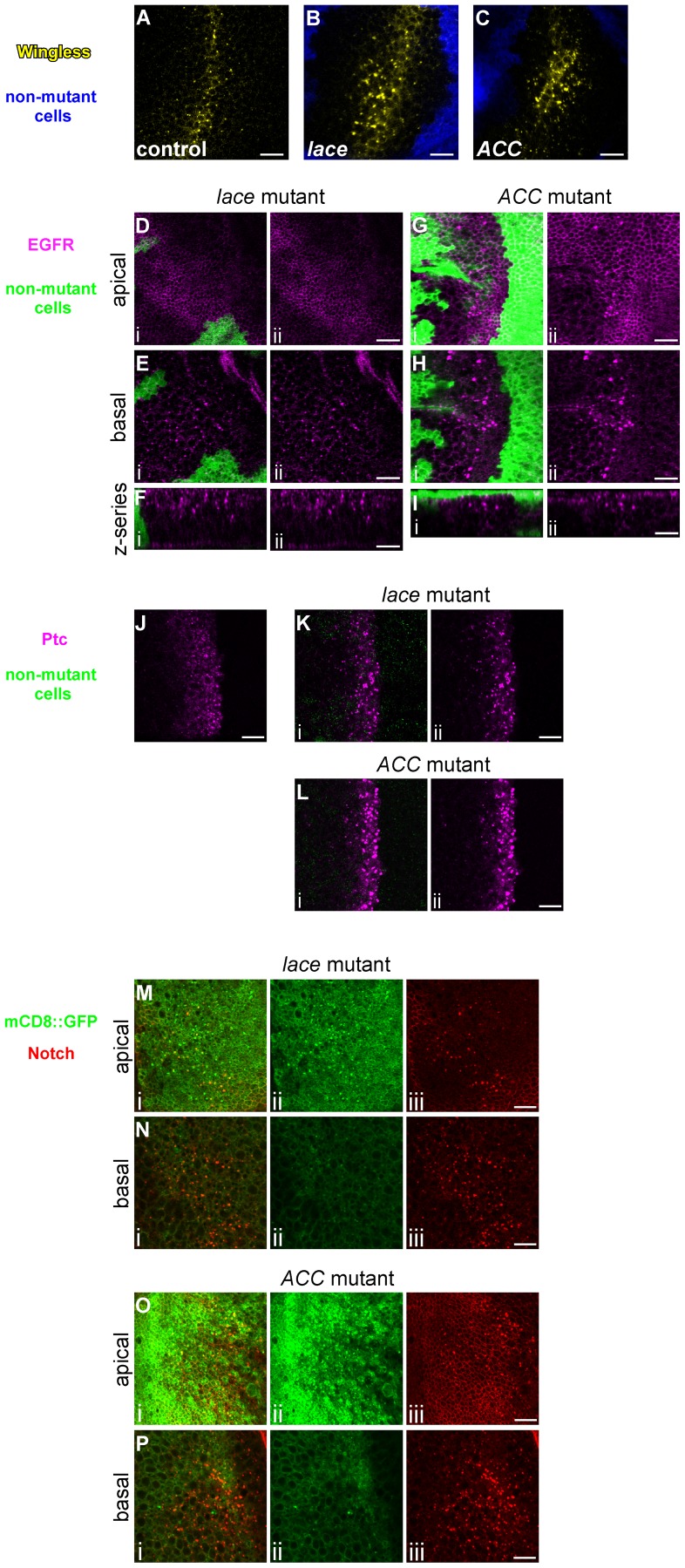
Abnormal trafficking of Wingless, EGFR, Patched, and mCD8::GFP in *lace* and *ACC* mutant tissues. Confocal optical sections through *D. melanogaster* wing imaginal discs bearing homozygous mutant clones of *lace^2^* (B, D–F, K, M, N) or *ACC^1^* (C, G–I, L, O, P) showing accumulation of Wingless (A–C), EGFR (D–I), Patched (J–L), and mCD8::GFP (M–P). For A–C, Wingless expression is shown in yellow, and homozygous mutant clone regions are indicated by the absence of blue marker signal. For D–I, K, and L, each image pair depicts (i) the merged signals showing protein accumulation (magenta) and clone locations (areas devoid of green GFP signal) and (ii) the protein accumulation signal alone. For M–P, image triplets depict (i) mCD8::GFP (green) and Notch (red) accumulation, (ii) mCD8::GFP alone, and (iii) Notch alone. Apical, basal, and vertical z-series orientations are indicated at left in D–I, M–P. Wildtype control images are shown for the endogenous Wingless (A) and Patched (J) non-uniform expression domains. Scale bars, 10 µm.

We examined the localization of additional cell-surface molecules to determine whether the requirement for *lace* and *ACC* is specific to Notch signaling or also pertains to other developmental pathways. Wingless, the secreted ligand of the Wnt pathway, the Epidermal Growth Factor Receptor (EGFR), a receptor tyrosine kinase that activates Ras/MAPK signaling, and Patched, the receptor for the Hedgehog signal, also accumulate non-cell-autonomously in large intracellular vesicles in *lace* or *ACC* homozygous mutant cells (Figure 2A–L). Moreover, expression of an exogenous mCD8::GFP fusion protein also leads to its weak overaccumulation in the enlarged Notch-associated vesicles in *lace* or *ACC* mutant cells, although this co-accumulation of Notch and mCD8:GFP is primarily observed in apical but not basal cell regions (Figure 2M–P). Thus *lace* and *ACC* are likely to play a general role in intracellular protein trafficking and may influence the proper routing of numerous membrane proteins.

### Molecular lesions in *lace* and *ACC*

**Figure 3:**
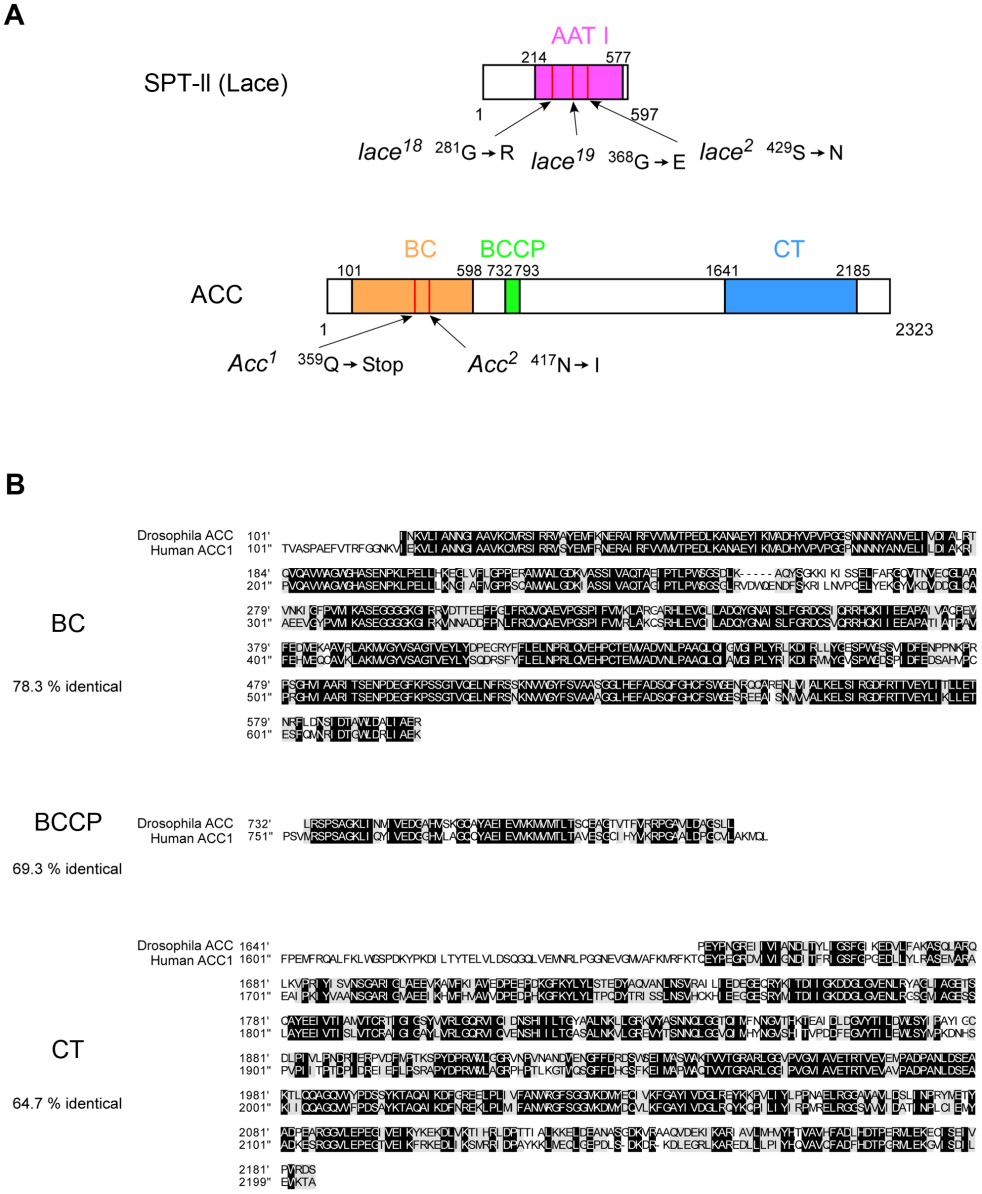
Analysis of molecular lesions associated with *lace* and *ACC* mutants and alignment of human and *D. melanogaster* ACC protein domains. (A) Diagram of the Lace protein (SPT-II, serine palmitoyltransferase II) showing amino acid substitutions in the AAT I (amino-acid acetyltransferase I) domain in *lace^18^*, *lace^19^*, and *lace^2^* mutants, and diagram of the ACC protein showing mutant lesions associated with *ACC^1^* and *ACC^2^* mutants. (B) Alignment of human and *D. melanogaster* ACC activity domains BC (biotin carboxylase), BCCP (biotin carboxyl carrier protein), and CT (carboxyltransferase) as depicted in panel A. Black boxes indicate identical residues; shaded boxes indicate conservative substitutions; percent identity is denoted at left for each domain.

Genetic mapping and complementation established that the newly recovered mutations are alleles of *lace*, which encodes serine palmitoyltransferase (SPT), and *ACC* encoding acetyl-CoA carboxylase. We determined the genomic sequence of the two *lace* alleles isolated from our screen as well as that of the previously isolated amorphic allele *lace^2^* [30], all of which bear point mutations altering a single amino acid (Figure 3A). As noted above, we confirmed that the null allele *lace^2^* shows the same non-cell-autonomous effect on Notch accumulation (see Supplemental Figure S1A–C), and thus *lace^2^* was utilized for all subsequent analyses.

The newly recovered *ACC* mutants fail to complement P-element insertion line B131, in which the transposon is inserted into the intron of *ACC* [31]. *ACC* encodes *D. melanogaster* acetyl-CoA carboxylase (ACC), an enzyme that is highly conserved from bacteria to humans [27]. Over three separate domains, >60% of the amino acids are identical between *D. melanogaster* ACC and human ACC1 (Figure 3B). We sequenced *ACC* mutant alleles *ACC^1^* and *ACC^2^*, and found that *ACC^1^* contains a premature stop codon, indicating that *ACC^1^* likely represents a null allele, while *ACC^2^* encodes an amino acid substitution within the N-terminal conserved domain (Figure 3A). For subsequent studies, we used the presumptive null allele *ACC^1^*.

To confirm that the Notch trafficking defects observed in these mutants are specifically attributable to loss of *lace* and *ACC* activity, we expressed wildtype *UAS-laceHA* [32] (in which Lace contains an HA-epitope tag) and *UAS-ACC* cDNA constructs in posterior compartment clones of *lace* and *ACC* mutant cells, respectively, utilizing a *hh-GAL4* driver line with *UAS-FLP* (see Materials and Methods). Expression of *UAS-laceHA* almost completely suppresses the Notch accumulation phenotype of *lace^2^* clones, and expression of *UAS-ACC* fully suppresses this phenotype in *ACC^1^* clones (Supplemental Figure S1F and S1G). To test whether non-specific *hh-GAL4*-mediated expression of either transgene can rescue the Notch accumulation defect, we also expressed *UAS-laceHA* in *ACC^1^* clones, and conversely *UAS-ACC* in *lace^2^* clones using the same approach. No significant rescue was observed in either case (Supplemental Figure S1H and S1I), indicating that the non-autonomous Notch trafficking defects seen in both mutants reflect specific requirements for *lace* and *ACC* gene activities rather than a general reduction in lipid homeostasis.

### Notch accumulates abnormally in endocytic compartments of mutant cells

To identify the cellular compartment in which Notch accumulates in the *lace* and *ACC* mutants, we performed antibody uptake studies on clone-bearing wing discs. When live, unpermeabilized discs are incubated with antibodies that bind to the extracellularly exposed domain of Notch, the antibodies specifically detect the subpool of Notch at the cell surface and in surface-derived endocytic compartments [33]. Incubating *lace* and *ACC* mosaic mutant discs with anti-Notch extracellular antibody C458.2H, followed by a brief incubation period to allow antibody uptake by the live cells, subsequent tissue fixation and imaging revealed that for both mutants, the abnormal Notch vesicles reside in the endocytic trafficking pathway (Figures 1B and 1E).

**Figure 4:**
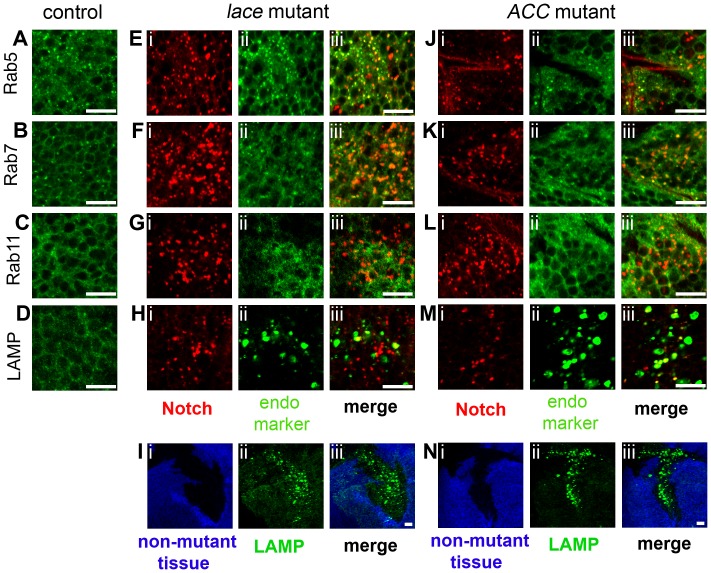
Colocalization of Notch with endosomal and lysosomal markers in *lace* and *ACC* mutant tissue clones. Each confocal image triplet (i–iii) depicts *lace^2^* (E–I) or *ACC^1^* (J–N) mutant wing disc clones, showing Notch overaccumulation (red in i for E–H, J–M) or mutant clone locations (absence of blue signal in i for I and N), the subcellular localization of the indicated organelle marker (green in ii), and the corresponding merged images at right (iii). Corresponding wildtype control images for each marker are shown in A–D; *da-GAL4; UAS-Rab5-YFP*, *Rab7-YFP*, or *Rab11-YFP* and *UAS-LAMP-HRP* were utilized for controls. Organelle markers in each panel are as follows: Rab5-YFP (Rab5; A, E, J), Rab7-YFP (Rab7; B, F, K), Rab11-YFP (Rab11; C, G, L), and LAMP-HRP (LAMP; D, H, I, M, N). Note elevated LAMP-HRP expression in *lace^2^* and *ACC^1^* mutant clones in I and N. Scale bars, 10 µm.

To determine the specific endocytic compartment in which Notch accumulates, we performed double-labeling studies with Notch and various organelle markers, including Rab5-YFP, Rab7-YFP, Rab11-YFP, and LAMP-HRP, which label early endosomes, late endosomes, recycling endosomes, and late endosomes/lysosomes, respectively [34], [35]. In *lace* mutant cells, 38% of abnormal Notch vesicles colocalize with Rab5-YFP, 16% with LAMP-HRP, and 11% with Rab7-YFP (Figure 4E–4H; Supplemental Table S1). In *ACC* mutant cells, 55% of Notch-positive vesicles colocalize with LAMP-HRP, 15% with Rab5-YFP, and 12% with Rab7-YFP (Figure 4J–4M; Supplemental Table S1). In both mutants, the Notch-positive vesicles exhibit a much lower degree of colocalization with other organelle markers, including Rab11-YFP, Clathrin light chain-GFP (Clc-GFP), PDI-GFP (an ER marker), Golgi-YFP, and Sara (Figure 4G and 4L; Supplemental Figure S2A–H; Supplemental Table S1). Interestingly, LAMP-positive late endosomes/lysosomes are dramatically enlarged in *lace* and *ACC* mutant cells (Figures 4I and 4N, Supplemental Table S2), while other compartments are only slightly affected (Supplemental Table S2). Collectively, these results indicate that Notch accumulates primarily in early endosomes, late endosomes and lysosomes in *lace* mutant cells, but predominantly in lysosomes, and to a lesser extent in early and late endosomes in *ACC* mutant cells. The distinct patterns of Notch mislocalization in the two mutants might reflect different alterations in these endocytic compartments, and might also contribute to the differential effects of these mutants on Notch and Wingless signaling (see below). However, it should be noted that these organelle marker studies involve overexpression of endosomal machinery components under *UAS* control, which although widely used to label different endocytic compartments, might lead to abnormal endosomal compartment morphogenesis and/or function.

### Loss of *lace* and *ACC* activity causes tissue overgrowth

**Figure 5:**
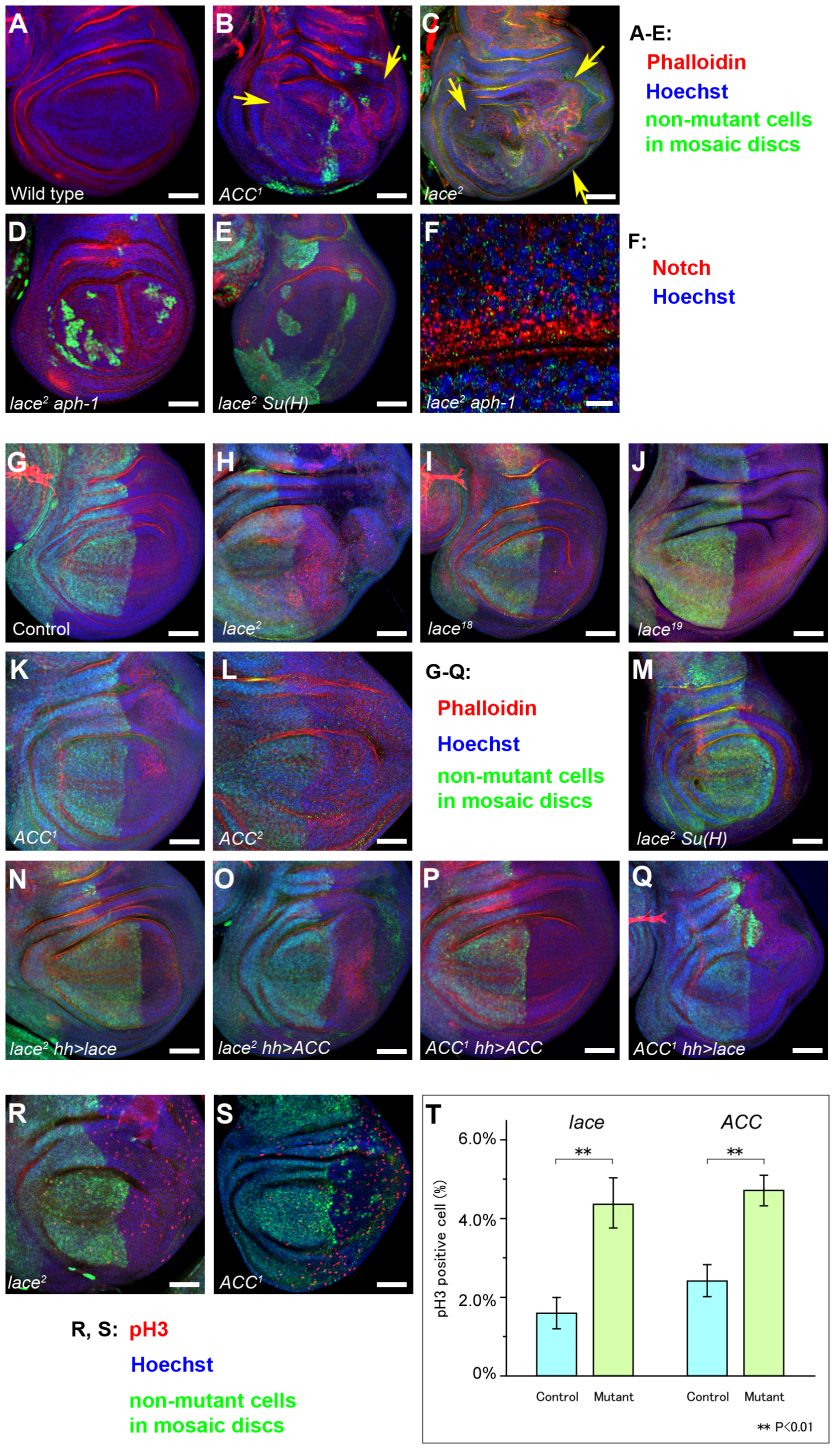
Analysis of tissue overgrowth and cell proliferation in *lace* and *ACC* mutant cells. Confocal images of wing disc pouches corresponding to (A) wildtype, (B) *ACC^1^* mutant clones, (C) *lace^2^* mutant clones (arrows indicate tissue overgrowth regions in B and C), (D) *lace^2^ aph-1^D35^* double mutant clones, and (E) *lace^2^ Su(H)^k07904^* double mutant clones, with confocal signals for Hoechst (blue), Phalloidin (red) and Myc (green) staining used to reveal tissue architecture and mutant vs. wildtype cell territories in clone-bearing discs. (F) Confocal section of a *lace^2^ aph-1^D35^* clone, showing high vesicular Notch accumulation (red). Hoechst-stained nuclei are shown in blue; faint green signal represents Myc antibody background staining that was used to identify the Myc-negative clones. (G–S) Mutant clones encompassing the wing posterior compartment were induced using the *hh-GAL4; UAS-FLP* system for (G) the control wildtype genotype (*FRT40A FRTG13*), (H, N, O, R) *lace^2^*, (I) *lace^18^*, (J) *lace^19^*, (K, P, Q, S) *ACC^1^*, (L) *ACC^2^*, and (M) *lace^2^ Su(H)^k07904^*. (N–Q) *UAS-cDNA* constructs encoding wildtype LaceHA (N, Q) or ACC (O, P) were expressed in either *lace^2^* (N, O) or *ACC^1^* (P, Q) mutant clones as indicated. Discs in G–Q were examined for Myc expression to identify Myc-negative clone regions (lack of green signal), Phalloidin (red), and Hoechst (blue). Genotypes of each panel correspond to those listed in Supplemental Table S3. (R, S) Wing imaginal discs with *lace^2^* (R) or *ACC^1^* (S) posterior compartment clones were analyzed with anti-phosphohistone H3 antibody (pH 3; red), anti-Myc (green; clone marker as above), and Hoechst (blue). Genotypes of (R) and (S) are the same as (H) and (K), respectively. (T) The percentages of pH 3-positive nuclei in *lace^2^* or *ACC^1^* homozygous mutant cells compared to control heterozygous cells were determined by analyzing Myc-negative and Myc-positive 127 µm×127 µm sectors, respectively, for ten discs of each genotype (**; P<0.01 by t-test). Scale bars, 50 µm in A–E, G–S; 10 µm in F.

The *lace* and *ACC* mutants were examined for whether they might also show a cell proliferation phenotype, since several *D. melanogaster* endocytic trafficking mutants cause cell overproliferation, especially in large clones produced using the *Minute* system [36] or through ectopic expression of the Caspase inhibitor p35 [10]–[14]. Using the *FLP-FRT* system with *Minute* chromosomes to generate large clones of either *lace* or *ACC* mutant cells, we found that these clones showed significant tissue overgrowth (Figure 5A–5C). To control for clone size and location, we next produced *lace* and *ACC* mutant clones in specific disc regions by expressing *UAS-FLP* under the control of *hh-GAL4*. Testing multiple alleles of *lace* and *ACC* using this approach revealed that the overgrowth phenotypes are variable, ranging from ∼2% up to ∼95% for different *lace* alleles, and from ∼10% to ∼28% for the two available *ACC* alleles (Figure 5G–L; Supplemental Table S3). The *lace* and *ACC* overproliferation phenotypes are almost completely suppressed by overexpression of Lace and ACC, respectively (Figure 5N and 5P; Supplemental Table S3). Consistent with our findings above for the Notch trafficking phenotype, the overproliferation phenotypes of *lace* and *ACC* mutant cells could not be rescued by the converse overexpression of ACC and Lace, respectively (Figure 5O and 5Q; Supplemental Table S3).

To confirm overproliferation at the cellular level in *lace* and *ACC* mutant cells, we examined phosphohistone H3 (pH 3) signals in clones of both mutants. The percentage of pH 3-positive cells is significantly increased in *lace* and *ACC* mutant cells compared to neighboring heterozygous cells (Figure 5R–T). We also examined apical-basal cell polarity in these overgrown mutant discs, since it is disrupted in other *D. melanogaster* endocytic mutants [10]–[14], in the glycosphingolipid metabolism mutants *egghead* and *brainiac* that disrupt Notch signaling during *D. melanogaster* oogenesis [37], and in several *C. elegans* mutants that likewise affect glycosphingolipid biosynthesis [38]. Unexpectedly, despite using four different markers to examine the apicobasal structure of *lace* and *ACC* mutant cells, we found that cell polarity is apparently unaffected in these mutant cells (Supplemental Figure S3A–T).

### Notch signaling is altered in *lace* and *ACC* mutant cells

**Figure 6:**
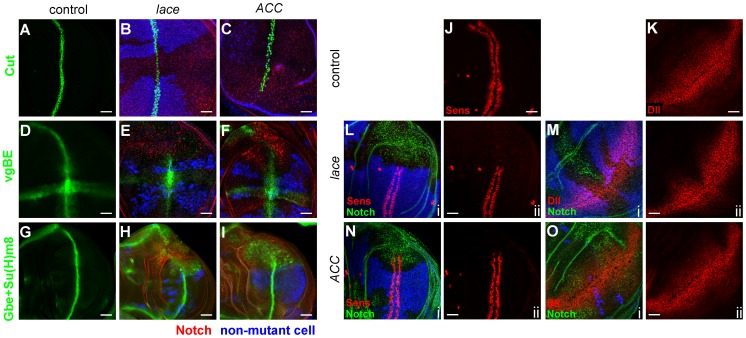
Notch and Wingless signaling abnormalities in *lace* and *ACC* mutants. (A–I) Wing disc mutant clones of *lace^2^* (B, E, H) and *ACC^1^* (C, F, I) analyzed for expression of the Notch pathway reporters Cut (B, C; green), *vestigial boundary enhancer* (E, F; vgBE; green), and *Gbe+Su(H)_m8_* (H, I; green). Mutant cell territories are indicated by absence of blue Myc or *lacZ* signal, and Notch accumulation is shown in red in B, C, E, F, H, and I. Wildtype expression patterns of the indicated Notch reporters are shown in A, D, and G (green). (J–O) Wing disc clones for *lace^2^* (L, M) and *ACC^1^* (N, O) were examined for activity of Wingless pathway reporters Senseless (Sens; J, L, N) and Distalless (Dll; K, M, O). For each panel i, mutant clone locations are indicated by absence of blue Myc or lacZ expression, Notch accumulation is shown in green, and Sens or Dll expression is in red; panel ii depicts the corresponding red channel only. Scale bars, 20 µm.

To determine which signaling pathways might be responsible for these overproliferation effects, we examined developmental gene expression patterns. Expression of Cut, which marks the presumptive wing margin during late larval development, was strongly reduced in regions where the margin extended deeply into *lace* or *ACC* mutant clones (Figure 6A–C). Cut expression depends on both Notch and Wingless activity [39]–[41], so we independently assessed Notch signaling in mutant clones using two additional Notch-responsive reporters. Expression of the *vestigial boundary enhancer-lacZ* (*vgBE-lacZ*) reporter [42] was diminished in *lace* and *ACC* mutant cells (Figure 6D–F). A second reporter, *Gbe+Su(H)_m8_* [43], also showed reduced expression along the dorsal-ventral (D/V) boundary in *lace* and *ACC* mutant clones; however, ectopic weak signal induction was also observed (Figure 6G–I). This result indicates that loss of either *lace* or *ACC* has complex, differential effects on Notch signal activation depending upon its cellular context.

These findings suggest that loss of *lace* or *ACC* has variable effects on Notch signaling in different tissues and even different wing disc regions. The *Gbe+Su(H)_m8_* expression data suggest that Notch signaling might be modestly upregulated in non-margin disc regions, potentially contributing to the overproliferation phenotype. To test this idea, we asked whether the overproliferation requires functional gamma-secretase, the proteolytic enzyme complex that cleaves Notch to produce NICD. Wing disc clones mutant for both *lace* and *aph-1* were generated, in which the *aph-1* mutation inactivates an essential subunit of gamma-secretase [44]. In these clones, the *aph-1* mutation strongly suppressed the overproliferation normally caused by the *lace* mutation but did not prevent elevated endosomal accumulation of Notch (Figure 5D and 5F). Taken together, these findings indicate that gamma-secretase-mediated Notch signaling activity is likely to be elevated in proliferating zones of the wing disc, leading directly or indirectly to the observed overgrowth. Confirming this interpretation, double mutant clones of *lace* and *Su(H)*, which encodes a dedicated effector for Notch signaling, also exhibited substantial suppression of the *lace* wing disc overgrowth phenotype (Figure 5E and 5M; Supplemental Figure S3).

### Wingless signaling is perturbed in *lace* and *ACC* mutants

**Figure 7:**
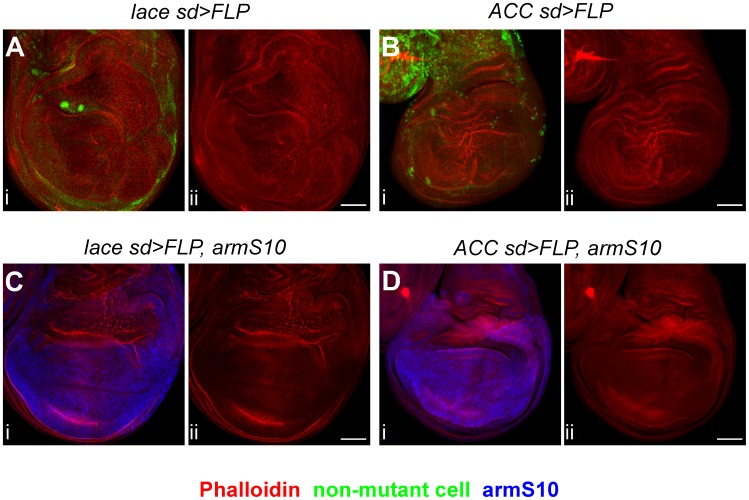
The *lace* overproliferation phenotype is partially rescued by activated Armadillo. Mutant clones of *lace^2^* (A, C) or *ACC^1^* (B, D) were induced using *sd-GAL4; UAS-FLP*, and examined for tissue overgrowth in the absence (A, B) or presence (C, D) of a constitutively activated form of Armadillo (armS10), expressed using a *UAS-armS10* transgene. For each image pair, panel i shows tissue growth patterns as revealed by Phalloidin (red in A–D), the mutant clone marker (green indicates non-mutant cells in A, B), or armS10 expression (blue in C, D), with the Phalloidin signal alone shown in panel ii. Complete genotypes are listed in Supplemental Table S4. Note that tissue overgrowth in the wing hinge region was not suppressed (C, D) because armS10 is not expressed in the hinge at this stage. Scale bars, 50 µm.

Wingless signaling is also altered in *lace* and *ACC* mutant cells based on expression of two downstream targets, Senseless (Sens) and Distalless (Dll), which respond to strong and weak Wingless signaling, respectively [41], [45], [46]. In *lace* mutant cells, expression of both Wingless targets is decreased (Figure 6L and 6M), while Sens expression is only weakly decreased and Dll expression is apparently unaffected in *ACC* mutant cells (Figure 6N and 6O). To determine whether altered Wnt signaling might contribute to the *lace* and *ACC* mutant overproliferation phenotypes, an activated form of *armadillo* (encoding *β*-Catenin, an effector of Wnt signal), termed *armS10*, was expressed in *lace* and *ACC* mutant clones. The overproliferation phenotype was significantly suppressed in *lace* mutant clones, in terms of both frequency and severity of the phenotype (Figure 7A and 7C; Supplemental Table S4). For *ACC* clones, the severity of the overgrowth phenotype was reduced by *armS10* expression (Figure 7B and 7D), although the percentage of discs exhibiting detectable overgrowth was not significantly different between control and *armS10*-expressing discs (Supplemental Table S4). These results are consistent with the idea that altered Wnt signaling partially contributes to the overproliferation phenotype in *lace* mutant cells, but seems to have a more modest role in the overproliferation observed for *ACC*-deficient cells.

In the above studies, we consistently observed that overgrowth phenotypes in *lace* and *ACC* clones were confined to the pouch and hinge region of the wing and not seen in the notum region, so we examined expression of a third Wnt downstream reporter, *fz3-lacZ*, in notum cells of mutant clone-bearing wing discs. Expression of *fz3-lacZ* was not altered in either *lace* or *ACC* mutant clones (data not shown), suggesting that the effects of the mutants on Wnt signaling, as with Notch signaling, are tissue-dependent.

### Altered Dpp, Hippo, MAPK, Akt and JAK-STAT signaling do not contribute to the overproliferation observed for *lace* and *ACC* mutant cells

We also examined two pathways that mediate cell proliferation in *D. melanogaster*, namely Dpp and Hippo signaling, to determine if their activity was also perturbed by loss of *lace* or *ACC* activity. In *lace* and *ACC* mutant clones, we failed to detect elevated or ectopic expression of phosphorylated Mad, which transduces the active Dpp signal [47], or Spalt, a transcriptionally-induced target of Dpp and Mad in the wing imaginal disc (Supplemental Figure S4C, S4D, S4G and S4H). In some large *lace* clones, expression of these markers was reduced or absent, which might be an indirect consequence of globally disrupted developmental patterning in large mutant clones (cf. Supplemental Figure S4C).

Two downstream markers of Hippo signaling, Cyclin E and *DIAP1-lacZ*, are upregulated when Hippo signaliing is inactivated and are also associated with overgrowth phenotypes [48]. We observed a slight increase in *DIAP1-lacZ* expression in *lace* and *ACC* clones (Supplemental Figure S4B and S4F), but no significant increase in Cyclin E expression (Supplemental Figure S4A and S4E), indicating that Hippo signaling makes little if any contribution to the cellular overproliferation seen in the mutants.

In addition, we assessed activation of other growth control pathways, including the EGFR, Insulin receptor, Hedgehog, and JAK/STAT pathways, by monitoring levels of their respective downstream markers dpERK, pAkt, Cubitus Interruptus, and pSTAT in *lace* and *ACC* mutant wing disc clones. Loss of *lace* or *ACC* function was not associated with any obvious disruptions in the levels or subcellular localizations of these pathway markers (Supplemental Figure S5A–L). These findings support the idea that Notch and Wnt signaling are relatively more sensitive to the loss of Lace and ACC enzyme activities, although due to the limitations of these antibody probes, we cannot exclude the possibility that some activities of these other pathways are also subtly perturbed.

## Discussion

The importance of lipid metabolism for the formation and maintenance of cell membranes is well established [24], [25], [49]. Both serine palmitoyltransferase (SPT) and acetyl-CoA carboxylase (ACC) are critical enzymes that control different steps of lipid metabolism, and are highly conserved in diverse animal species. Genetic elimination of ACC1 or the SPT subunits Sptlc1 or Sptlc2 cause early embryonic lethality in mice [50], [51], although the cellular basis for this lethality is unknown. In *D. melanogaster*, RNA-interfering disruption of ACC activity in the fat body results in reduced triglyceride storage and increased glycogen accumulation, and in oenocytes leads to loss of watertightness of the tracheal spiracles causing fluid entry into the respiratory system [52]. Here we demonstrate that *D. melanogaster* mutants lacking functional SPT or ACC exhibit endosomal trafficking defects, causing Notch, Wingless, EGFR, and Patched to accumulate abnormally in endosomes and lysosomes. These effects are accompanied by significant alterations in Notch and Wingless signaling, as revealed by changes in downstream target gene activation for both pathways. However, the mutants do not fully inactivate these developmental signaling pathways, and instead display phenotypes consistent with more complex, pleiotropic effects on Notch, Wingless, and potentially additional pathways in different tissues. Our findings reinforce the importance of lipid metabolism for the maintenance of proper developmental signaling, a concept that has also emerged from studies demonstrating that *D. melanogaster* mutants for *phosphocholine cytidylyltransferase* alter endosomal trafficking and signaling of Notch and EGFR [15], mutants for *alpha*-*1,4-N-acetylgalactosaminyltransferase-1* affect endocytosis and activity of the Notch ligands Delta and Serrate [16], mutants for the ceramide synthase gene *shlank* disrupt Wingless endocytic trafficking and signaling [21], and mutants for the glycosphingolipid metabolism genes *egghead* and *brainiac* modify the extracellular gradient of the EGFR ligand Gurken [53].

Most strikingly, our newly characterized *lace* and *ACC* mutants also display prominent tissue overgrowth phenotypes. These tissue overgrowth effects are linked to changes in Notch and Wingless signaling outputs, and they involve gamma-secretase, Su(H), and Armadillo activities, suggesting that the overgrowth reflects an interplay of Wingless inactivation and Notch hyperactivation. Consistent with our findings, both Notch and Wingless regulate cell proliferation and imaginal disc size in *D. melanogaster* [54]. Moreover, several observations indicate that Notch and Wingless are jointly regulated by endocytosis, with opposing effects on their respective downstream pathway activities, a dynamic process that might be especially sensitive to perturbations in membrane lipid constituents [55]. Wingless itself exerts opposing effects on disc size that might depend on the particular developmental stage or disc territory. For example, hyperactivation of Wingless or inactivation of its negative regulators cause overproliferation [56]–[58], but Wingless activity can also constrain wing disc growth [59]. Similar spatiotemporal effects might underlie the variability we detected in our studies with *lace* and *ACC* mutant clones, in which both tissue overgrowth and developmentally arrested discs were observed. Although we did not detect obvious changes in downstream signaling for several other cell growth pathways that were examined, the trafficking abnormalities seen for other membrane proteins aside from Notch, Delta, and Wingless, as well as the incomplete suppression of the overgrowth phenotypes by blockage of Notch and Wingless signaling, suggest that other pathways might also be dysregulated in *lace* and *ACC* mutants, possibly contributing to the observed tissue overgrowth.

Wingless is modified by lipid addition [19], [20], and lipoprotein vesicles have been suggested to control Wingless diffusion [60]. In *D. melanogaster* embryos, endocytosis of Wingless limits its diffusion and ability to act as a long-range morphogen [61]. Endocytosis can also affect Wingless signaling in receiving cells, where endocytosis both promotes signal downregulation [61], [62] and positively facilitates signaling [63]. The apparently normal diffusion ranges for overaccumulated Wingless in *lace* and *ACC* mutant clones, yet reduced downstream target gene expression, is consistent with the idea that SPT and ACC act by promoting endocytic trafficking of Wingless in receiving cells rather than influencing the secretion and/or diffusion of Wingless from signal-sending cells.

Our finding that *lace* and *ACC* mutant overgrowth phenotypes are also partially Notch-dependent is reminiscent of similar overproliferation phenotypes seen in certain *D. melanogaster* endocytic mutants, such as *vps25*, and *tsg101* [11]–[14]. The overproliferation of disc tissue in these mutants is attributable to Notch hyperactivation, reflecting the fact that non-ligand-bound Notch receptors that are normally targeted for recycling or degradation are instead retained and signal from endosomes. Analogous effects are likely to contribute to the *lace* and *ACC* mutant overgrowth, where we observe significant Notch overaccumulation throughout the endosomal-lysosomal routing pathway. Some ectopic Notch signaling might emanate from the lysosomal compartment, which is enlarged and accumulates particularly high levels of Notch in *lace* and *ACC* mutant clones. Analysis of *D. melanogaster HOPS* and *AP-3* mutants, which affect protein delivery to lysosomes, has identified a lysosomal pool of Notch that is able to signal in a ligand-independent, gamma-secretase-dependent manner [64].

How do SPT and ACC contribute to endosomal trafficking of Notch and other proteins? In the yeast SPT mutant *lcb1*, an early step of endocytosis is impaired due to defective actin attachment to endosomes, a phenotype that is suppressed by addition of sphingoid base [65]. However, the trafficking abnormalities seen in *lace* and *ACC* mutants do not resemble those in the yeast *lcb1* mutant, perhaps because endocytic vesicle fission is primarily dependent upon dynamin in *D. melanogaster* and mammals, instead of actin as in yeast [66]. Nevertheless, the requirement for SPT and ACC in *D. melanogaster* endosomal compartments might reflect possible functions in endosome-cytoskeleton interactions. Another possibility is that the defective endosomal trafficking seen in *lace* and *ACC* mutants is caused by the inability to synthesize specific phospholipids needed for normal membrane homeostasis. Finally, *lace* and *ACC* might be important for the formation and/or function of lipid rafts, specialized membrane microdomains that have been implicated in both signaling and protein trafficking [67], [68].

A remarkable feature of the *lace* and *ACC* mutant phenotypes that suggests an underlying defect in lipid biogenesis is the non-autonomous effect in mutant tissue clones, wherein nearby wildtype cells generate a secreted activity that diffuses several cell diameters into the mutant tissue and rescues the trafficking and signaling defects. One possibility is that these secreted activities are diffusible lipid biosynthetic products of SPT and ACC, which enter the mutant cells and serve as precursors for further biosynthetic steps that do not require SPT or ACC. An intriguing alternative is that the SPT and ACC enzymes are themselves secreted and taken up by the mutant cells. A precedent for this mechanism has recently been demonstrated for *D. melanogaster* ceramidase, a sphingolipid metabolic enzyme that is secreted extracellularly, delivered to photoreceptors, and internalized by endocytosis to regulate photoreceptor cell membrane turnover [69].

Recent work has highlighted the importance of lipid metabolism for oncogenic transformation, and ACC has been advanced as a promising target for cancer drug development [70]. ACC is upregulated in some cancers, possibly as a result of high demands for lipid biosynthesis during rapid cell divisions. Sphingolipids and their derivatives are also thought to influence the balance of apoptosis and cell proliferation during tissue growth, and thus have also garnered attention as potential cancer therapy targets [71]. Our findings regarding the requirements of SPT and ACC for proper trafficking and signaling of key developmental cell-surface signaling molecules, including Notch and Wingless, provide insights into how lipid metabolic enzymes might influence cell proliferation and tissue patterning in multicellular animals. Complex lipid biosynthesis is essential for the creation of the elaborate, interconnected, and highly specialized membrane compartments in which developmental pathways operate, and perturbations in lipid biosynthesis that are tolerated by the cell might nevertheless exert significant pleiotropic effects on developmental patterning, cell proliferation, and other cellular processes. Exploration of lipid metabolic enzymes as pharmacological targets must therefore take into account potentially unfavorable effects on critical signaling pathways controlling development and organogenesis.

## Materials and Methods

### Constructs

A full-length *ACC* cDNA (GH12002; obtained from the *Drosophila* Genomics Resource Center, Indiana University) was subcloned into the *XbaI* and *SmaI* sites of pBluescript II SK^−^, introducing a new *NotI* site between *KpnI* and *HindIII*. The resulting *NotI* fragment was excised and inserted into the *NotI* site of the *pUAST* vector. Transformants of *UAS-ACC* were obtained according to standard protocols.

### *D. melanogaster* genetics

Mutagenesis was performed using standard protocols by administering 35 mM ethylmethanesulfonate to isogenic male flies of genotype *y w; P{ry[+t7.2] = neoFRT}40A P{w[+mW.hs] = FRT(w^hs^)}G13*, which were used to establish candidate mutant stocks. For screening of 3335 mutagenized second chromosome arms, these stocks were mated to marked 2L and 2R FRT stocks to yield progeny bearing homozygous candidate mutant wing clones using the FLP/FRT method [29]. 10 wing discs of each candidate mutant line were harvested and analyzed for abnormal Notch accumulation by direct immunofluorescence using Notch antibody C17.9C6 as described below.

*D. melanogaster* stocks used included Oregon-R as wild type, *th^j5C8^* as a *DIAP1-lacZ* marker (Bloomington *Drosophila* Stock Center), *UAS-Rab5-YFP*, *UAS-Rab7-YFP*, *UAS-Rab11-YFP* [34], *UAS-LAMP-HRP* [35], *UAS-EGFP-clc* [72], *sqh-EYFP-Golgi* [73], and *PDI-GFP* [74] as intracellular compartment markers, *UAS-laceHA* [32] as a *lace* rescue transgene, *UAS-armS10* [75] to express activated Armadillo, *vgBE-lacZ* [42] and *Gbe+Su(H)_m8_* [43] as Notch target gene reporters, *da-GAL4* and *hh-GAL4* (courtesy of Dr. Jin Jiang) as GAL4 drivers, *fz3-lacZ* (*fz3^J29^* [76]) as a Wingless signal reporter, *P{Ubi-GFP(S65T)nls}2L FRT40A*, *M(2)24F^1^ P{πM}36F FRT40A*, *P{arm-lacZ.V}36BC FRT40A*, *FRTG13 P{Ubi-GFP.nls}2R1 P{Ubi-GFP.nls}2R2*, *FRT42D P{πM}45F M(2)53^1^*, and *FRT42D P{arm-lacZ.V}51D* as FLP-FRT clone makers, with *P{hsFLP}12* or *P{UAS-FLP.Exel}1* as FLP sources.

Double mutant clones of *lace^2^ aph-1^D35^* and *lace^2^ Su(H)^k07904^* were produced by the FLP/FRT method following recombination of *aph-1^D35^* [44] and *Su(H)^k07904^* [77] onto the *FRT40A lace^2^* chromosome. To check Notch intracellular localization, *hs-FLP; lace^2^ FRT40A/tub-GAL80 FRT40A; da-GAL4* combined with either *UAS*-driven *Rab5-YFP*, *7-YFP*, *11-YFP*, *EGFP-clc*, or *LAMP-HRP*, or *hs-FLP; FRTG13 ACC^1^/FRTG13 tub-GAL80 da-GAL4* combined with either *UAS*-driven *Rab5-YFP*, *7-YFP*, *11-YFP*, *EGFP-clc*, or *LAMP-HRP* larvae were dissected and stained with anti-Notch and anti-GFP antibodies described below.

### Immunohistology

Wing imaginal discs were dissected, fixed, and immunostained [44] using the following primary antibodies: mouse Notch intracellular domain antibody C17.9C6 (1∶1000; [78]; DSHB, University of Iowa); mouse Notch extracellular domain antibody C458.2H (1∶500; [79]; DSHB, University of Iowa); rat Notch3 (1∶1000; courtesy of Dr. Spyros Artavanis-Tsakonas); mouse Delta antibody C594.9B (1∶1000; [80]; DSHB, University of Iowa); mouse Cut 2B10 (1∶1000; DSHB, University of Iowa); rat ELAV antibody 7E8A10 (1∶500; DSHB, University of Iowa); mouse Wg antibody 4D4 (1∶500; [81]; DSHB, University of Iowa); guinea pig Sens antibody (1∶1000; [45]; courtesy of Dr. Hugo Bellen); mouse Distalless antibody DMDll.1 (1∶400; [82]; courtesy of Dr. Ian Duncan); mouse Engrailed antibody 4D9 (1∶500; [83]; DSHB, University of Iowa); rat CycE antibody (1∶1000; [84]; courtesy of Dr. Helena Richardson); rabbit phosphorylated Mad antibody PS1 (1∶500; [47]; courtesy of Dr. Carl H. Heldin); rabbit Spalt antibody (1∶400; [85]; courtesy of Dr. Rosa Barrio); rabbit Sara antibody (1∶500; [86]; courtesy of Dr. Franck Coumailleau); rat DE-Cadherin antibody DCAD2 (1∶20; [87]; DSHB, University of Iowa); mouse Discs large 4F3 (1∶10; [88]; DSHB, University of Iowa); rabbit PKCξ antibody C20 (1∶1000; Santa Cruz); mouse Armadillo antibody N2 7A1 (1∶500; [89]; DSHB, University of Iowa); mouse CD2 antibody MCA154GA (1∶1000; AbD Serotech); chicken Myc antibody NB600-334 (1∶1000; Novus Biologicals); rat Myc antibody JAC6 (1∶500; Novus Biologicals); rabbit GFP antibody 598 (1∶1000; MBL); rat GFP antibody GF090R (1∶500; NacalaiTesque); mouse ß-galactosidase antibody Z378A (1∶1000; Promega); chicken ß-galactosidase antibody XW-7591 (1∶1000; ProSci); mouse HRP antibody 2H11 (1∶500; Santa Cruz Biotechnology); goat anti-Egfr antibody dC-20 (1∶500; Santa Cruz Biotechnology), mouse anti-Patched antibody (1/500; [90]; DSHB, University of Iowa), mouse anti-Active(dp) MAPK antibody A3713 (1∶500; Sigma), rabbit anti-phospho-*Drosophila* Akt (Ser505) Antibody (Cell Signaling Technology), rat anti-Cubitus Interruptus 2A1 (1/500; [91]; DSHB, University of Iowa), rabbit anti-phosphohistone H3 Ser10 antibody (Upstate), rabbit anti-phosphorylated STAT (Cell Signaling Technology). Confocal images were acquired using LSM510META and LSM700 (Zeiss) confocal microscopes, and fluorescent intensity was measured using ImageJ software.

For live tissue labeling, dissected wing discs were incubated with antibody for 40 min in S2 cell culture medium (Gibco), washed three times for 10 min with S2 medium, fixed and processed further as above. Hoechst-33342 trihydrochloride trihydrate (Invitrogen; 1∶1000 dilution) and Phalloidin-Alexa546 (Molecular Probes; 1∶20 dilution) stainings were performed for 1 hr at room temperature following immunostaining.

## Supporting Information

Figure S1 Notch accumulates abnormally in homozygous tissue clones mutant for different *lace* and *ACC* alleles, and this phenotype is rescued by corresponding wildtype *lace* or *ACC* transgene expression. (A–E) Confocal sections through *D. melanogaster* wing imaginal discs bearing homozygous *lace^2^* (A–C), *lace^19^* (D), and *ACC^2^* (E) mutant clones, depicting apical (A) and basal (B, D, E) horizontal sections and a vertical z-series image compilation (C). For each image pair, panel i shows mutant clone locations (areas devoid of green signal) and Notch protein distribution (red); the Notch signal alone is presented in panel ii. (F–I) Mutant clones encompassing the wing posterior compartment were induced as in Figure 5G–S using the *hh-GAL4; UAS-FLP* system for *lace^2^* (F, H) or *ACC^1^* (G, I), where clones also expressed either *UAS-laceHA* (F, I) or *UAS-ACC* (G, H) wildtype cDNA transgenes as indicated. Note that expression of *UAS-laceHA* rescues the *lace^2^* Notch trafficking defect, and conversely, expression of *UAS-ACC* rescues the *ACC^1^* Notch trafficking defect, but neither transgene rescues the Notch trafficking defects seen in mutant clones for the non-matching gene. In F–I, mutant cells are identified by their lack of green marker signal, and Notch expression is shown in red. Scale bars, 10 µm.
(TIF)

Figure S2 Lack of colocalization of Notch with certain organelle markers in *lace* and *ACC* mutant tissues. Each confocal image triplet (i–iii) depicts *lace^2^* (A–D) or *ACC^1^* (E–H) mutant wing disc clones, showing Notch overaccumulation (red in i), subcellular localization of the indicated organelle marker (green in ii), and the corresponding merged images at right (iii) with mutant clone regions indicated by absence of blue signal in panel iii for B–D and F–H. For A and E, *lace^2^* and *ACC^1^* mutant clones were identified by the clone-specific expression of Clathrin light chain-GFP using the MARCM technique (see Materials and Methods). Organelle markers are indicated at left and are as follows: Clathrin light chain-EGFP (Clc; A, E), Sara endosomes (B, F), Spaghetti squash-EYFP-Golgi (Golgi; C, G), and PDI-GFP (D, H). Scale bars, 10 µm.
(TIF)

Figure S3 Apicobasal cell polarity is not significantly altered in *lace* and *ACC* mutant tissues. Posterior wing disc compartment clones mutant for *lace^2^* (A–E, K–O) or *ACC^1^* (F–J, P–T) were produced using *hh-GAL4; UAS-FLP* and analyzed with antibodies recognizing aPKC (A–J, green), Armadillo (arm; A–J, red), DE-cadherin (DE-Cad; K–T, green), and Discs large (Dlg; K–T, red). Blue signal corresponds to the Myc marker used to identify heterozygous cells; mutant clones are identified by absence of this marker. Heterozygous control (white boxes) and homozygous mutant (yellow boxes) tissue sectors of the discs in A, F, K, and P are shown at higher magnification in B–E, G–J, L–O, and Q–T, respectively, with control cells in B, C, G, H, L, M, Q and R, and mutant cells in D, E, I, J, N, O, S, and T, as indicated at center. Apical horizontal (B, D, G, I, L, N, Q, S) and vertical z-series (C, E, H, J, M, O, R, T) optical sections are presented for these high-magnification images. Each image triplet (i–iii) includes the merged three-channel image (i), the isolated green channel image (ii), and the isolated red channel image (iii). Scale bars, 20 µm.
(TIF)

Figure S4 Expression of cell proliferation pathway markers in *lace* and *ACC* mutants. Wing disc clones mutant for *lace^2^* (A–D) or *ACC^1^* (E–H) were examined for expression of Cyclin E (CycE; A, E), DIAP1-*lacZ* (DIAP; B, F), phosphorylated Mad (pMad; C, G), or Spalt (D, H). Each image triplet (i–iii) includes (i) overlay of the confocal channels showing Notch accumulation (red) and mutant (absence of blue Myc signal) versus non-mutant control (blue Myc signal) tissue regions, (ii) overlay of all three confocal channels showing Notch (red), mutant versus control cell territories (blue), and expression of the relevant marker protein Cyclin E, DIAP1, pMad, or Spalt (green; marker proteins indicated at left), and (iii) marker protein only. Scale bars, 50 µm.
(TIF)

Figure S5 EGFR, Insulin Receptor, Hedgehog, and JAK-STAT signaling are not hyperactivated in *lace* and *ACC* mutant clones. Wing imaginal discs lacking homozygous mutant clones (control; A, D, G, J), or containing *lace^2^* (B, E, H, K) or *ACC^1^* (C, F, I, L) mutant clones were analyzed using antibodies that recognize active MAPK (dpERK; A–C), phosphorylated Akt (pAkt; D–F), Cubitus interruptus (Ci; G–I), or phosphorylated STAT (pSTAT; J–L), as shown in green and indicated at left. For each mutant image pair (i–ii) in B, C, E, F, H, I, K, and L, panel i shows clone locations (areas devoid of blue marker signal) superimposed on the activated pathway component signal (green), and panel ii shows the isolated green channel signal alone. Scale bars, 50 µm.
(TIF)

Table S1 Quantitative analysis of Notch vesicle colocalization with specific organelle markers in *lace* and *ACC* mutant cells. Homozygous *lace^2^* (top) or *ACC^1^* (bottom) mutant clones were generated in wing imaginal discs, which were analyzed using Notch antibodies together with several specific organelle markers (listed at left). Confocal z-series optical sections encompassing the entire apicobasal extent of each clone were scored for the total number of enlarged Notch-positive vesicles detected (right column) and the percentage of these Notch-positive vesicles that were co-labeled by a given organelle marker (middle column).
(DOC)

Table S2 Intensity difference of specific organelle markers between control and *lace* or *ACC* mutant cells. Confocal horizontal optical sections of *lace^2^* (top) or *ACC^1^* (bottom) mutant clones were compared to non-mutant control cells by measuring the average fluorescence intensity difference of several organelle markers (listed at left) between equivalently sized sectors of mutant and control tissue. Sector sizes were 403.58 µm^2^ for *PDI-GFP* and *Golgi-YFP*, 408.64 µm^2^ for *lamp-HRP*, or 100.89 µm^2^ for Sara, *Clc-GFP*, *Rab11-YFP*, *Rab5-YFP*, and *Rab7-YFP*. 10 confocal optical sections were measured for each genotype/organelle marker combination; numerical data are mean intensity +/− standard deviation.
(DOC)

Table S3 Quantitative analysis of tissue overgrowth phenotypes in *lace* and *ACC* mutant clone-bearing wing discs. Summary of overgrowth phenotypes exhibited by different genotypes used in this study. Leftmost column lists the primary mutant and/or transgenic genotypes, followed by columns showing the percentages of clone-bearing wing discs exhibiting overgrowth, the total numbers of wing discs examined, and the full genotypes used to generate the mutant clones and/or transgene expression for each sample.
(DOC)

Table S4 Quantitative analysis of cellular overproliferation phenotypes in *lace* and *ACC* mutant clones expressing activated Armadillo. Summary of overgrowth phenotypes exhibited by *lace^2^* and *ACC^1^* mutant clones in the absence or presence of constitutively activated Armadillo, produced under control of *sd-GAL4; UAS-FLP*. Leftmost column specifies the mutant clone genotypes (*lace^2^ sd>FLP* or *ACC^1^ sd>FLP*) and whether or not activated Armadillo was also expressed in the mutant clones (*armS10*), followed by columns showing the percentages of clone-bearing wing discs exhibiting overgrowth, the total numbers of wing discs examined, and the full genotypes used to generate the mutant clones and/or transgene expression for each sample.
(DOC)
