# Supplementary figures and images for: Disruption of Drosophila melanogaster Lipid Metabolism Genes Causes Tissue Overgrowth Associated with Altered Developmental Signaling
Source: PLoS Genet. 2013 Nov 7;9(11):e1003917. doi: 10.1371/journal.pgen.1003917 (PMC3820792; doi:10.1371/journal.pgen.1003917)

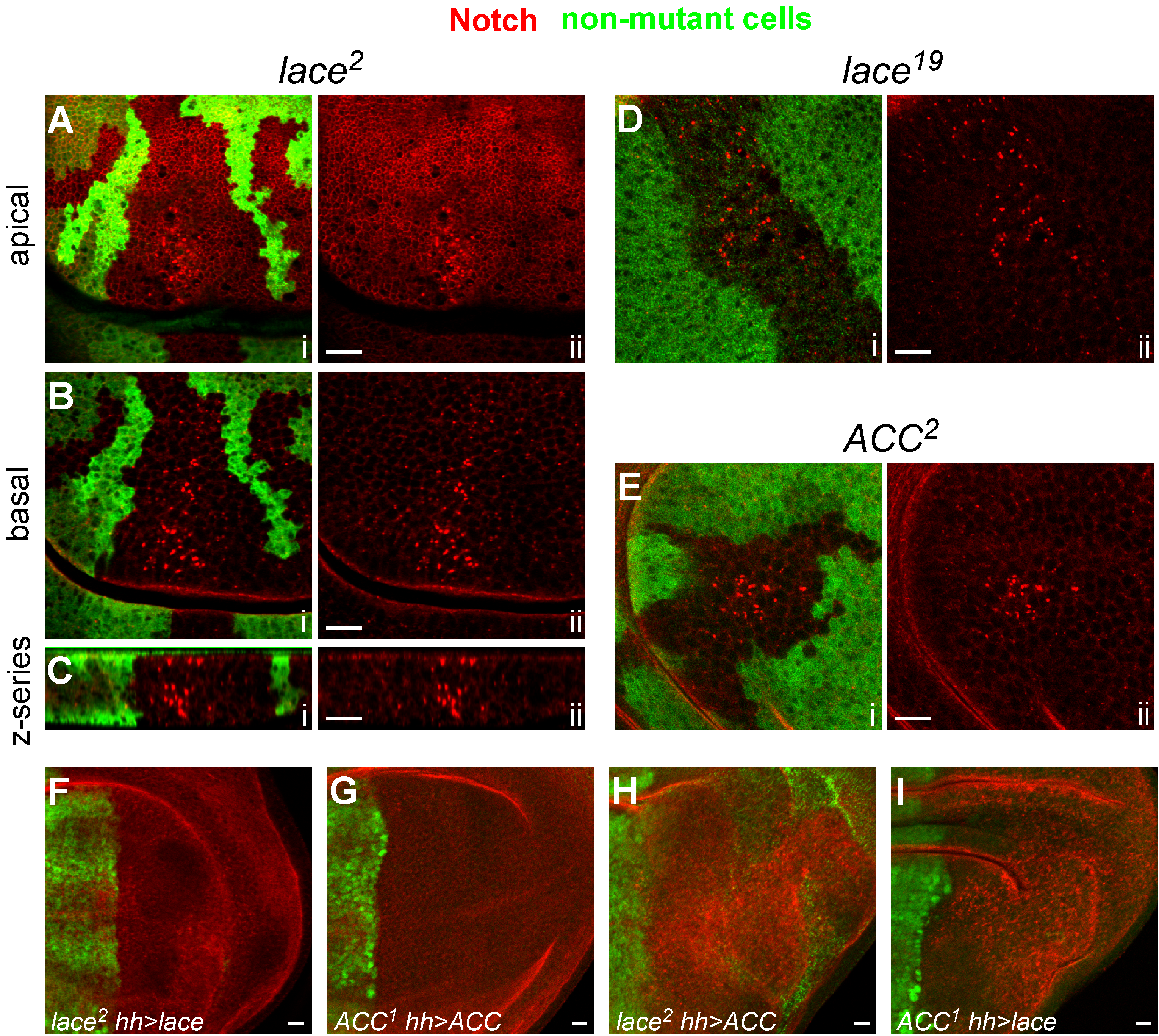

Supplement: Figure S1 — Notch accumulates abnormally in homozygous tissue clones mutant for different lace and ACC alleles, and this phenotype is rescued by corresponding wildtype lace or ACC transgene expression. (A–E) Confocal sections through D. melanogaster wing imaginal discs bearing homozygous lace2 (A–C), lace19 (D), and ACC2 (E) mutant clones, depicting apical (A) and basal (B, D, E) horizontal sections and a vertical z-series image compilation (C). For each image pair, panel i shows mutant clone locations (areas devoid of green signal) and Notch protein distribution (red); the Notch signal alone is presented in panel ii. (F–I) Mutant clones encompassing the wing posterior compartment were induced as in Figure 5G–S using the hh-GAL4; UAS-FLP system for lace2 (F, H) or ACC1 (G, I), where clones also expressed either UAS-laceHA (F, I) or UAS-ACC (G, H) wildtype cDNA transgenes as indicated. Note that expression of UAS-laceHA rescues the lace2 Notch trafficking defect, and conversely, expression of UAS-ACC rescues the ACC1 Notch trafficking defect, but neither transgene rescues the Notch trafficking defects seen in mutant clones for the non-matching gene. In F–I, mutant cells are identified by their lack of green marker signal, and Notch expression is shown in red. Scale bars, 10 µm. (TIF) [file pgen.1003917.s001.tif]

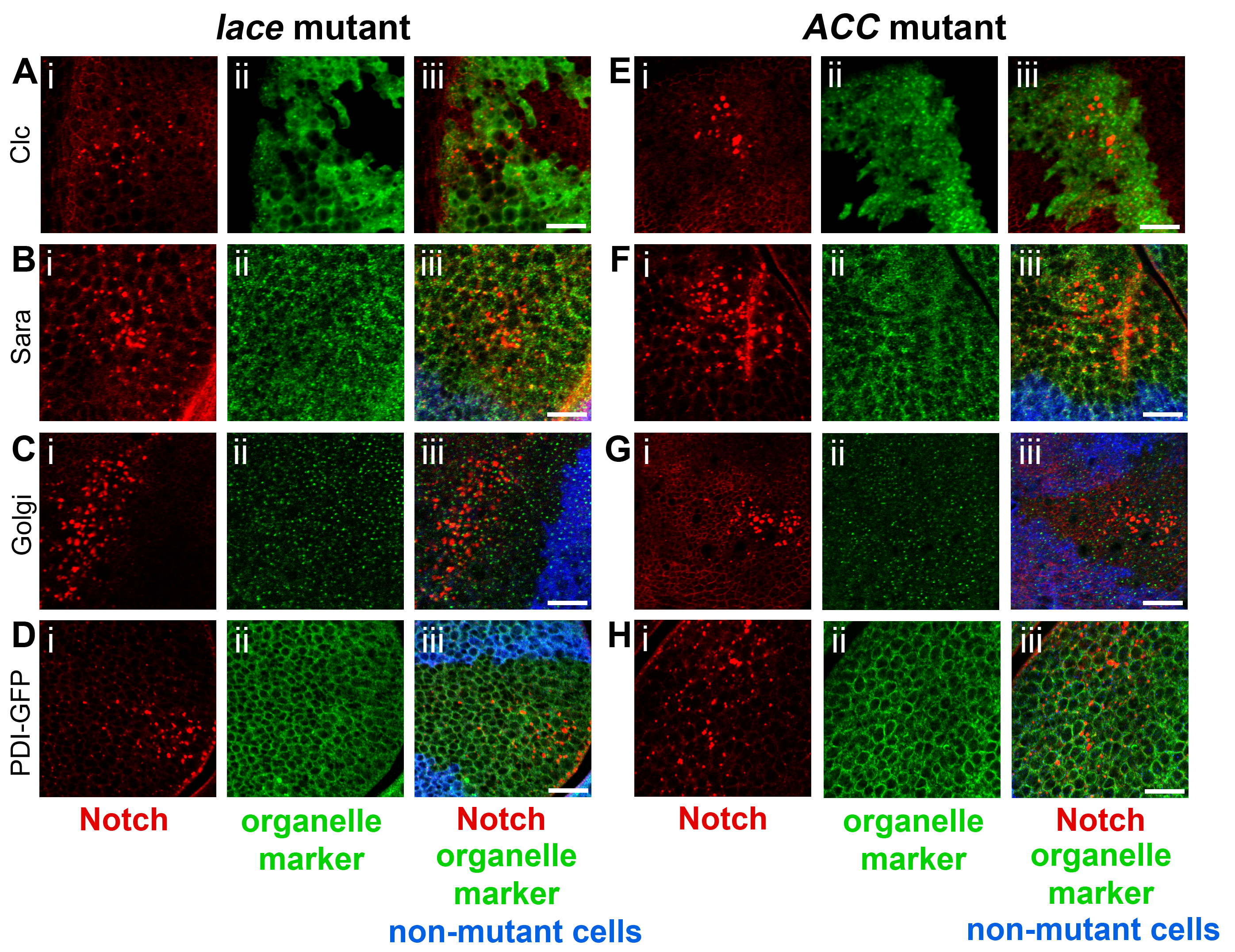

Supplement: Figure S2 — Lack of colocalization of Notch with certain organelle markers in lace and ACC mutant tissues. Each confocal image triplet (i–iii) depicts lace2 (A–D) or ACC1 (E–H) mutant wing disc clones, showing Notch overaccumulation (red in i), subcellular localization of the indicated organelle marker (green in ii), and the corresponding merged images at right (iii) with mutant clone regions indicated by absence of blue signal in panel iii for B–D and F–H. For A and E, lace2 and ACC1 mutant clones were identified by the clone-specific expression of Clathrin light chain-GFP using the MARCM technique (see Materials and Methods). Organelle markers are indicated at left and are as follows: Clathrin light chain-EGFP (Clc; A, E), Sara endosomes (B, F), Spaghetti squash-EYFP-Golgi (Golgi; C, G), and PDI-GFP (D, H). Scale bars, 10 µm. (TIF) [file pgen.1003917.s002.tif]

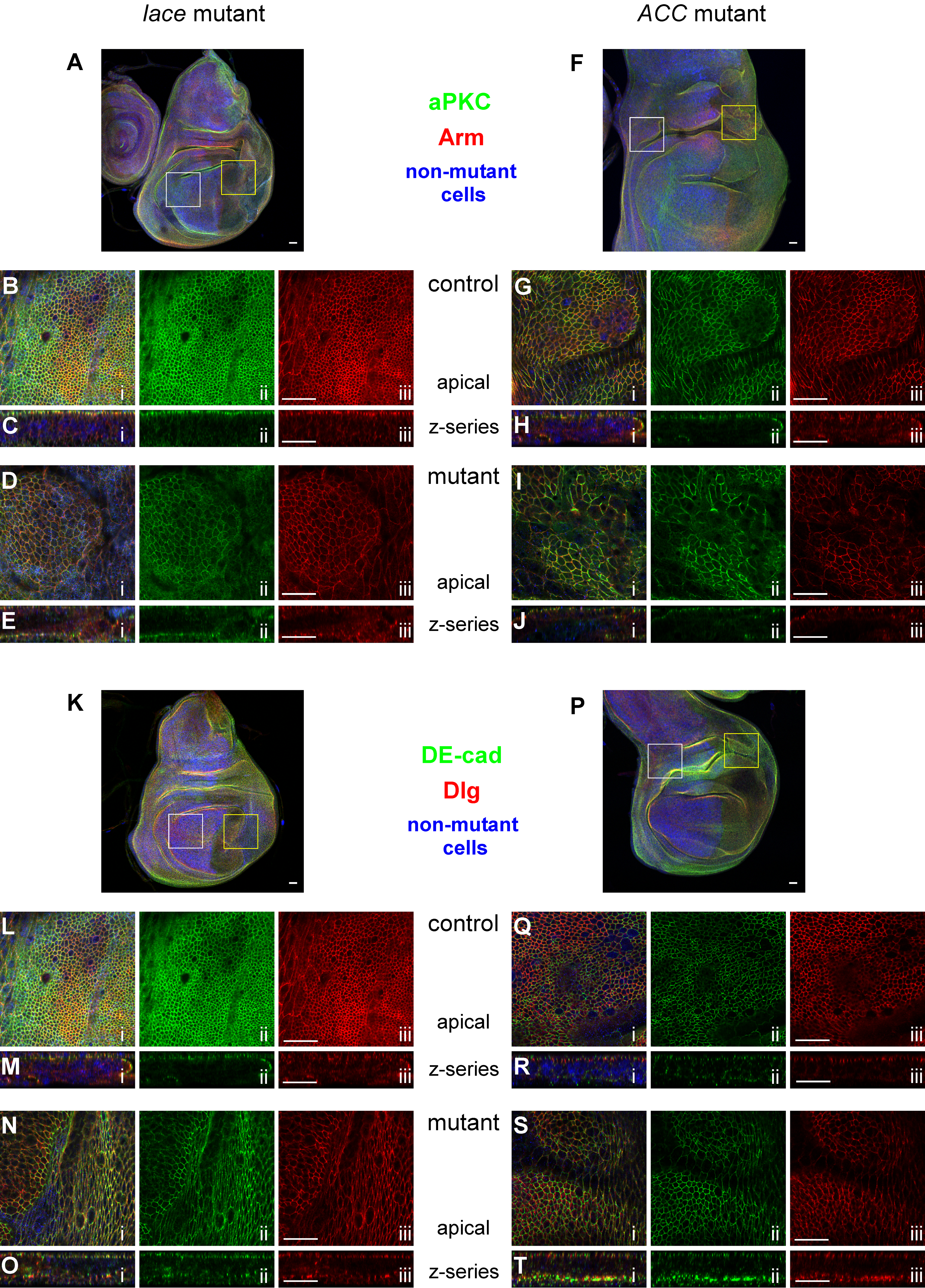

Supplement: Figure S3 — Apicobasal cell polarity is not significantly altered in lace and ACC mutant tissues. Posterior wing disc compartment clones mutant for lace2 (A–E, K–O) or ACC1 (F–J, P–T) were produced using hh-GAL4; UAS-FLP and analyzed with antibodies recognizing aPKC (A–J, green), Armadillo (arm; A–J, red), DE-cadherin (DE-Cad; K–T, green), and Discs large (Dlg; K–T, red). Blue signal corresponds to the Myc marker used to identify heterozygous cells; mutant clones are identified by absence of this marker. Heterozygous control (white boxes) and homozygous mutant (yellow boxes) tissue sectors of the discs in A, F, K, and P are shown at higher magnification in B–E, G–J, L–O, and Q–T, respectively, with control cells in B, C, G, H, L, M, Q and R, and mutant cells in D, E, I, J, N, O, S, and T, as indicated at center. Apical horizontal (B, D, G, I, L, N, Q, S) and vertical z-series (C, E, H, J, M, O, R, T) optical sections are presented for these high-magnification images. Each image triplet (i–iii) includes the merged three-channel image (i), the isolated green channel image (ii), and the isolated red channel image (iii). Scale bars, 20 µm. (TIF) [file pgen.1003917.s003.tif]

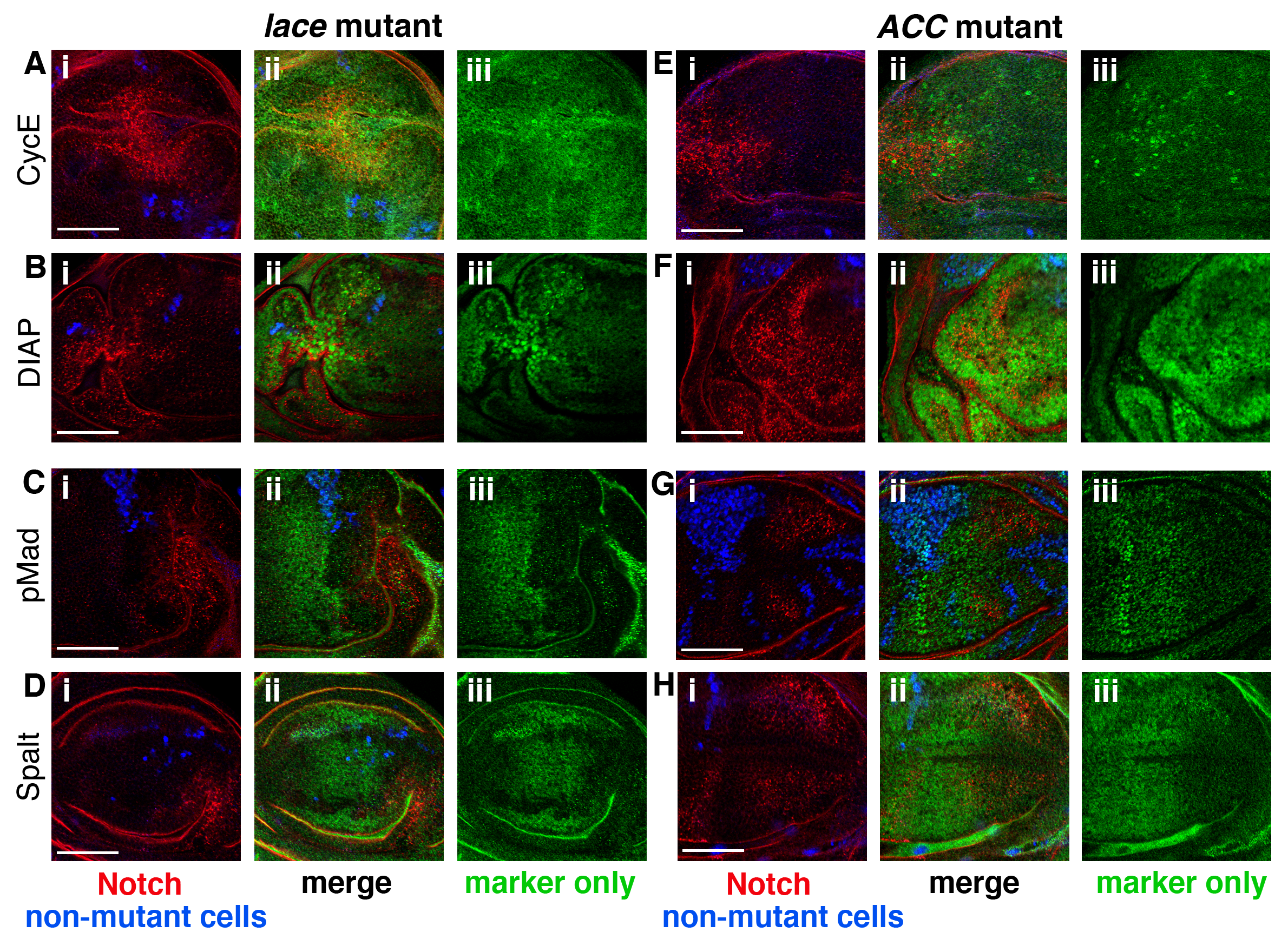

Supplement: Figure S4 — Expression of cell proliferation pathway markers in lace and ACC mutants. Wing disc clones mutant for lace2 (A–D) or ACC1 (E–H) were examined for expression of Cyclin E (CycE; A, E), DIAP1-lacZ (DIAP; B, F), phosphorylated Mad (pMad; C, G), or Spalt (D, H). Each image triplet (i–iii) includes (i) overlay of the confocal channels showing Notch accumulation (red) and mutant (absence of blue Myc signal) versus non-mutant control (blue Myc signal) tissue regions, (ii) overlay of all three confocal channels showing Notch (red), mutant versus control cell territories (blue), and expression of the relevant marker protein Cyclin E, DIAP1, pMad, or Spalt (green; marker proteins indicated at left), and (iii) marker protein only. Scale bars, 50 µm. (TIF) [file pgen.1003917.s004.tif]

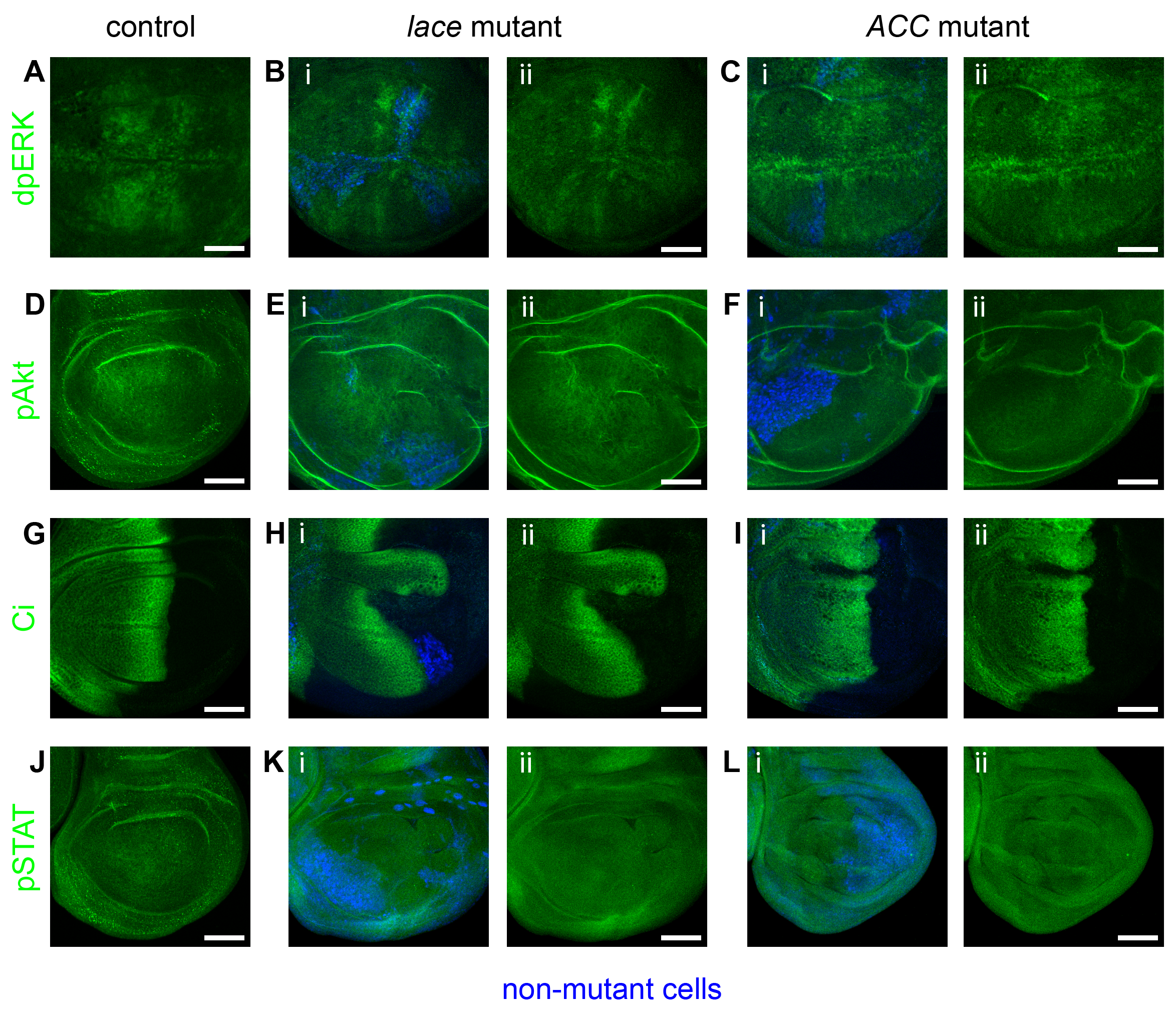

Supplement: Figure S5 — EGFR, Insulin Receptor, Hedgehog, and JAK-STAT signaling are not hyperactivated in lace and ACC mutant clones. Wing imaginal discs lacking homozygous mutant clones (control; A, D, G, J), or containing lace2 (B, E, H, K) or ACC1 (C, F, I, L) mutant clones were analyzed using antibodies that recognize active MAPK (dpERK; A–C), phosphorylated Akt (pAkt; D–F), Cubitus interruptus (Ci; G–I), or phosphorylated STAT (pSTAT; J–L), as shown in green and indicated at left. For each mutant image pair (i–ii) in B, C, E, F, H, I, K, and L, panel i shows clone locations (areas devoid of blue marker signal) superimposed on the activated pathway component signal (green), and panel ii shows the isolated green channel signal alone. Scale bars, 50 µm. (TIF) [file pgen.1003917.s005.tif]
