# Supplementary material for: Disruption of Drosophila melanogaster Lipid Metabolism Genes Causes Tissue Overgrowth Associated with Altered Developmental Signaling
Source: PLoS Genet. 2013 Nov 7;9(11):e1003917. doi: 10.1371/journal.pgen.1003917 (PMC3820792; doi:10.1371/journal.pgen.1003917)
Supplement: Table S1 — Quantitative analysis of Notch vesicle colocalization with specific organelle markers in lace and ACC mutant cells. Homozygous lace2 (top) or ACC1 (bottom) mutant clones were generated in wing imaginal discs, which were analyzed using Notch antibodies together with several specific organelle markers (listed at left). Confocal z-series optical sections encompassing the entire apicobasal extent of each clone were scored for the total number of enlarged Notch-positive vesicles detected (right column) and the percentage of these Notch-positive vesicles that were co-labeled by a given organelle marker (middle column). (DOC) [file pgen.1003917.s006.doc]

Table S1. Sasamura et al.

|  | percentage of marker-positive enlarged Notch vesicles | total number of enlarged Notch vesicles scored |
| --- | --- | --- |
|  |  |  |
| ***lace^2^*** |  |  |
|  |  |  |
| *PDI-GFP* | 0.55 | 731 |
| *Golgi-YFP* | 0.78 | 509 |
| *Clc-GFP* | 0.97 | 310 |
| Sara | 1.9 | 592 |
| *Rab11-YFP* | 0.57 | 883 |
| *Rab5-YFP* | 38 | 1689 |
| *Rab7-YFP* | 11 | 584 |
| *Lamp-HRP* | 16 | 1327 |
|  |  |  |
| ***ACC^1^*** |  |  |
|  |  |  |
| *PDI-GFP* | 0.19 | 527 |
| *Golgi-YFP* | 1.6 | 654 |
| *Clc-GFP* | 0.69 | 583 |
| Sara | 1.1 | 571 |
| *Rab11-YFP* | 0.76 | 654 |
| *Rab5-YFP* | 15 | 1555 |
| *Rab7-YFP* | 12 | 1139 |
| *Lamp-HRP* | 55 | 327 |
