# Supplementary material for: Disruption of Drosophila melanogaster Lipid Metabolism Genes Causes Tissue Overgrowth Associated with Altered Developmental Signaling
Source: PLoS Genet. 2013 Nov 7;9(11):e1003917. doi: 10.1371/journal.pgen.1003917 (PMC3820792; doi:10.1371/journal.pgen.1003917)
Supplement: Table S2 — Intensity difference of specific organelle markers between control and lace or ACC mutant cells. Confocal horizontal optical sections of lace2 (top) or ACC1 (bottom) mutant clones were compared to non-mutant control cells by measuring the average fluorescence intensity difference of several organelle markers (listed at left) between equivalently sized sectors of mutant and control tissue. Sector sizes were 403.58 µm2 for PDI-GFP and Golgi-YFP, 408.64 µm2 for lamp-HRP, or 100.89 µm2 for Sara, Clc-GFP, Rab11-YFP, Rab5-YFP, and Rab7-YFP. 10 confocal optical sections were measured for each genotype/organelle marker combination; numerical data are mean intensity +/− standard deviation. (DOC) [file pgen.1003917.s007.doc]

Table S2. Sasamura et al.

|  | marker intensity  (arbitrary units) |
| --- | --- |
|  |  |
| ***lace^2^*** |  |
|  |  |
| *PDI-GFP* | 36.0 ±35.1 |
| *Golgi-YFP* | 3.13 ±2.75 |
| *lamp-HRP* | 1460 ±307 |
| Sara | 26.1 ±21.6 |
|  |  |
| *Clc-GFP* | 0.848 ±0.508 |
| *Rab11-YFP* | 0.873 ±0.703 |
| *Rab5-YFP* | 1.80 ±1.06 |
| *Rab7-YFP* | 2.01 ±0.667 |
|  |  |
| ***ACC^1^*** |  |
|  |  |
| *PDI-GFP* | 27.0 ±25.0 |
| *Golgi-YFP* | 2.67 ±2.42 |
| *lamp-HRP* | 2196 ±226 |
| Sara | 12.5 ±13.8 |
|  |  |
| *Clc-GFP* | 1.18 ±0.876 |
| *Rab11-YFP* | 2.07 ±1.82 |
| *Rab5-YFP* | 2.75 ±1.41 |
| *Rab7-YFP* | 3.63 ±1.54 |
