# Supplementary material for: Disruption of Drosophila melanogaster Lipid Metabolism Genes Causes Tissue Overgrowth Associated with Altered Developmental Signaling
Source: PLoS Genet. 2013 Nov 7;9(11):e1003917. doi: 10.1371/journal.pgen.1003917 (PMC3820792; doi:10.1371/journal.pgen.1003917)
Supplement: Table S3 — Quantitative analysis of tissue overgrowth phenotypes in lace and ACC mutant clone-bearing wing discs. Summary of overgrowth phenotypes exhibited by different genotypes used in this study. Leftmost column lists the primary mutant and/or transgenic genotypes, followed by columns showing the percentages of clone-bearing wing discs exhibiting overgrowth, the total numbers of wing discs examined, and the full genotypes used to generate the mutant clones and/or transgene expression for each sample. (DOC) [file pgen.1003917.s008.doc]

Table S3. Sasamura et al.

| clone genotype | percentage (%) of wing discs with overgrowth | number of wing discs examined | full genotype used to produce mutant clones |
| --- | --- | --- | --- |
|  |  |  |  |
| Control | 0.0 | 191 | *UAS-FLP/+; FRT40A FRTG13/ RpL27A πM FRT40A; hh-GAL4/+* |
| *lace^2^* | 95.0 | 199 | *UAS-FLP/+; lace^2^ FRT40A / RpL27A πM FRT40A; hh-GAL4/+* |
| *lace^18^* | 1.9 | 211 | *UAS-FLP/+; lace^18^ FRT40A / RpL27A πM FRT40A; hh-GAL4/+* |
| *lace^19^* | 26.9 | 26* | *UAS-FLP/+; lace^19^ FRT40A / RpL27A πM FRT40A; hh-GAL4/+* |
| *ACC^1^* | 28.4 | 183 | *UAS-FLP/+;FRT42D ACC^1^/FRT42D πM M(2)53; hh-GAL4/+* |
| *ACC^2^* | 10.8 | 212 | *UAS-FLP/+;FRT42D ACC^2^/FRT42D πM M(2)53; hh-GAL4/+* |
| *lace^2^ hh>lace* | 2.5 | 121 | *UAS-FLP/+; lace^2^ FRT40A / RpL27A πM FRT40A; hh-GAL4/UAS-laceHA* |
| *lace^2^ hh>ACC* | 87.1 | 116 | *UAS-FLP/+; lace^2^ FRT40A / RpL27A πM FRT40A; hh-GAL4/UAS-ACC* |
| *ACC^1^ hh>lace* | 24.1 | 145 | *UAS-FLP/+;FRT42D ACC^1^/FRT42D πM M(2)53; hh-GAL4/UAS-laceHA* |
| *ACC^1^ hh>ACC* | 0.0 | 187 | *UAS-FLP/+;FRT42D ACC^1^/FRT42D πM M(2)53; hh-GAL4/UAS-ACC* |
| *lace^2^ Su(H)^k07904^* | 38.7 | 31* | *UAS-FLP/+; Su(H) ^k07904^ lace^2^ FRT40A / RpL27A πM FRT40A; hh-GAL4/+* |

* Due to poor stock viability, only a small number of wing imaginal discs were recovered for this genotype
