# Supplementary material for: Disruption of Drosophila melanogaster Lipid Metabolism Genes Causes Tissue Overgrowth Associated with Altered Developmental Signaling
Source: PLoS Genet. 2013 Nov 7;9(11):e1003917. doi: 10.1371/journal.pgen.1003917 (PMC3820792; doi:10.1371/journal.pgen.1003917)
Supplement: Table S4 — Quantitative analysis of cellular overproliferation phenotypes in lace and ACC mutant clones expressing activated Armadillo. Summary of overgrowth phenotypes exhibited by lace2 and ACC1 mutant clones in the absence or presence of constitutively activated Armadillo, produced under control of sd-GAL4; UAS-FLP. Leftmost column specifies the mutant clone genotypes (lace2 sd>FLP or ACC1 sd>FLP) and whether or not activated Armadillo was also expressed in the mutant clones (armS10), followed by columns showing the percentages of clone-bearing wing discs exhibiting overgrowth, the total numbers of wing discs examined, and the full genotypes used to generate the mutant clones and/or transgene expression for each sample. (DOC) [file pgen.1003917.s009.doc]

Table S4. Sasamura et al.

| clone genotype | percentage (%) of wing discs with overgrowth | number of wing discs examined | full genotype used to produce mutant clones |
| --- | --- | --- | --- |
|  |  |  |  |
| *lace^2^ sd>FLP* | 95.2 | 227 | *sd-GAL4/+; lace^2^ FRT40A / RpL27A πM FRT40A; UAS-FLP/+* |
| *ACC^1^ sd>FLP* | 13.7 | 190 | *sd-GAL4/+;FRT42D ACC^1^/FRT42D πM M(2)53; UAS-FLP/+* |
| *lace^2^ sd>FLP, armS10* | 45.5 | 165 | *sd-GAL4/UAS-FLP UAS-armS10; lace^2^ FRT40A / RpL27A πM FRT40A* |
| *ACC^1^ sd>FLP, armS10* | 12.0 | 217 | *sd-GAL4/UAS-FLP UAS-armS10;FRT42D ACC^1^/FRT42D πM M(2)53* |
